# A New Design of the Dual-Mode and Pure Longitudinal EMAT by Using a Radial-Flux-Focusing Magnet

**DOI:** 10.3390/s22041316

**Published:** 2022-02-09

**Authors:** Xu Zhang, Weiwen Li, Bo Li, Jun Tu, Chunhui Liao, Qiao Wu, Sheng Feng, Xiaochun Song

**Affiliations:** 1 Hubei Key Laboratory of Modern Manufacturing Quantity Engineering, School of Mechanical Engineering, Hubei University of Technology, Wuhan 430068, China; zhangxu@mail.hbut.edu.cn (X.Z.); 102010061@hbut.edu.cn (W.L.); 102110151@hbut.edu.cn (B.L.); juntu@hbut.edu.cn (J.T.); 20161010@hbut.edu.cn (C.L.); qiao_wu@hbut.edu.cn (Q.W.); 2 Xianning Institute of Information and Standardization, Xianning 437000, China; 101810129@hbut.edu.cn

**Keywords:** electromagnetic acoustic transducer, longitudinal wave, flux-concentrating, transducer design

## Abstract

An electromagnetic acoustic transducer (EMAT) is suitable for measuring the propagation time more accurately without causing abrasion to the transducer during testing due to the principle of its excitation. This work designs a flux-concentrating EMAT with a radial-flux-focusing permanent magnet to significantly enhance static magnetic field strength. Through theoretical analysis and finite element simulation, two kinds of coils are designed according to the concentration areas of the horizontal and vertical components of the magnetic field. One is used to generate pure longitudinal waves, and the other is used to generate both longitudinal waves and shear waves. The experimental comparison shows that the amplitudes of the pure longitudinal wave and the dual-mode wave excited by the two kinds of coils with the radial-flux-focusing magnet are more than two times higher than those with the ordinary magnet. Therefore, the flux-concentrating EMAT with the appropriate coil provides an insight into realizing more accurate detection where longitudinal wave detection is required.

## 1. Introduction

Electromagnetic acoustic transducers (EMATs) are widely used in the field of nondestructive testing (NDT), and have the superiority of non-contact and no couplant with the specimen [1]. Unlike EMATs, the couplant is needed between the piezoelectric ultrasonic transducer and the specimen surface, so the elastic waves propagate not only in the specimen but also in the couplant and the transducer. Thus, the reflection echoes received by piezoelectric transducers are accompanied by interference echoes from the inside of the transducer and the interface of the couplant, which affects the calculation of the ultrasonic propagation time interval in the received signal [2]. However, the ultrasonic wave source excited by EMATs is inside the specimen, which is more suitable for measuring the propagation time accurately without causing abrasion to the transducer during testing [3]. At present, shear wave excitation is mainly used in EMATs for thickness measurement, flaw detection and defect detection in many industrial fields [4,5,6,7]. Due to the structural characteristics of shear wave EMATs [8,9,10,11], shear waves are easily generated and received. Longitudinal-wave EMATs have inefficient energy conversion because of the low horizontal magnetic field intensity required to generate longitudinal waves and the sizeable parasitic inductance of the coil [12,13]. However, the longitudinal-wave EMATs have a broad application prospect. Since the velocity of longitudinal waves is nearly twice that of shear waves, the longitudinal waves excited by electromagnetic ultrasound are more efficient in the detection of the thickness of large aluminum plates and other similar metals. Furthermore, the longitudinal waves combined with the shear waves excited by the EMAT probe could take advantage of contactless detection in measurements of stress and elastic constants [14,15,16,17,18].

At present, there are few structures of longitudinal-wave EMATs. There are three typical designs: Hirao and Ogi [19] pointed out that a bulk wave EMAT that consists of a single-cylindrical permanent magnet and a spiral coil can generate longitudinal waves and radially polarized shear waves. Da Cunha and Jordan [20] proposed a longitudinal-wave EMAT consisting of a cylindrical permanent magnet and external magnetic rings, with an iron coupling between the internal magnetic rod and outer magnetic rings to provide a stronger horizontal magnetic field. Wu et al. [21] used a combination of a large-diameter center magnet and a ring magnet to enhance the strength of the horizontal magnetic field, and a sheet of copper was placed between the coil and the specimen to control the eddy current distribution, which aims to suppress shear waves. Considering the Lorentz forces as the dominant transduction mechanism, increasing the strength of the static magnetic field plays an important role in increasing ultrasonic wave amplitude. The optimization of the magnet arrangement has a significant effect on improving the static magnetic field [8,9,22,23]. In particular, the Halbach structure is better than the traditional soft iron backing for enhancing the static magnetic field and achieved good results in dual-mode excitation [24].

In this paper, a radial-flux-focusing magnet is proposed, inspired by Halbach’s concept, which can provide a stronger magnetic field, thus improving the efficiency of the energy transfer of the longitudinal-wave EMAT and making the measurement signal easier to identify. This work also utilizes two kinds of spiral coils [25] by changing their size parameters according to the location of the area where the horizontal and vertical components of the magnetic field are concentrated. When dual modes are needed, the amplitude of shear waves and longitudinal waves simultaneously excited by the flux-concentrating EMAT can be increased using the large-diameter coil. When a pure longitudinal wave is needed, the amplitude of shear waves excited by the flux-concentrating EMAT with the small-diameter coil decreases, while the amplitude of longitudinal waves increases.

## 2. Configuration and Operating Principle of the Proposed Flux-Concentrating EMAT

**Figure 1:**
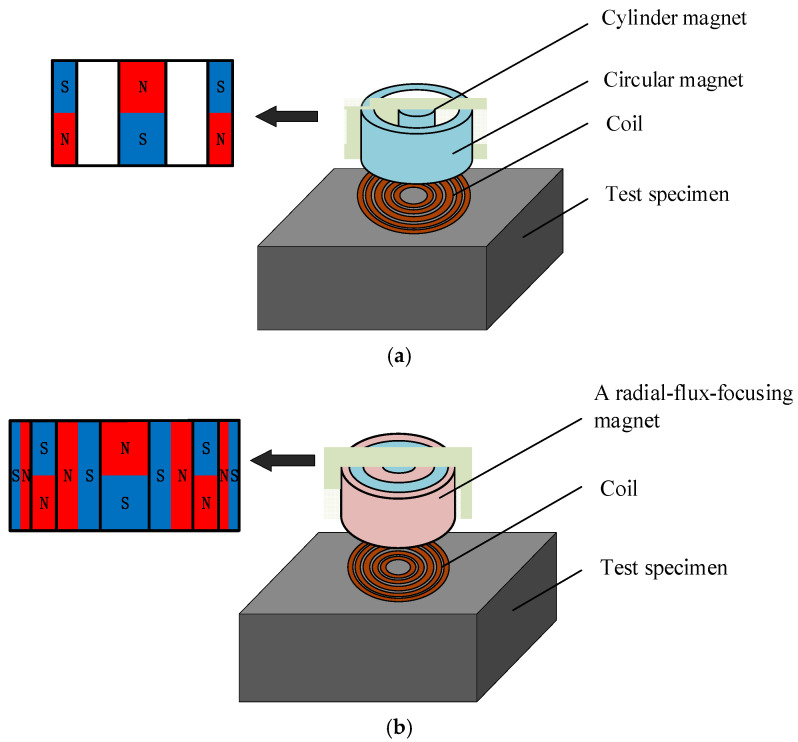
Configuration of (**a**) the ring-type EMAT and (**b**) the flux-concentrating EMAT.

The structure diagram of the ring-type EMAT is shown in Figure 1a. The permanent magnets of the transducer include a cylinder magnet in the middle and a surrounding circular magnet, and the gap between the two magnets is filled with epoxy resin. The poles of the circular-ring permanent magnet and the cylindrical permanent magnet are opposite at the same ends. Therefore, a horizontal radial magnetic field is distributed between the two magnets. Figure 1a shows a typical longitudinal-wave EMAT used as a comparison in this article since its structure is similar to the designed EMAT. Figure 1b shows the configuration of the flux-concentrating EMAT. A radial-magnetized annular magnet is filled between the cylinder magnet and the surrounding circular magnet, and another radial-magnetized annular magnet is also covered outside the surrounding circular magnet. The magnetizing directions of the magnets are all clearly illustrated in Figure 1.

**Figure 2:**
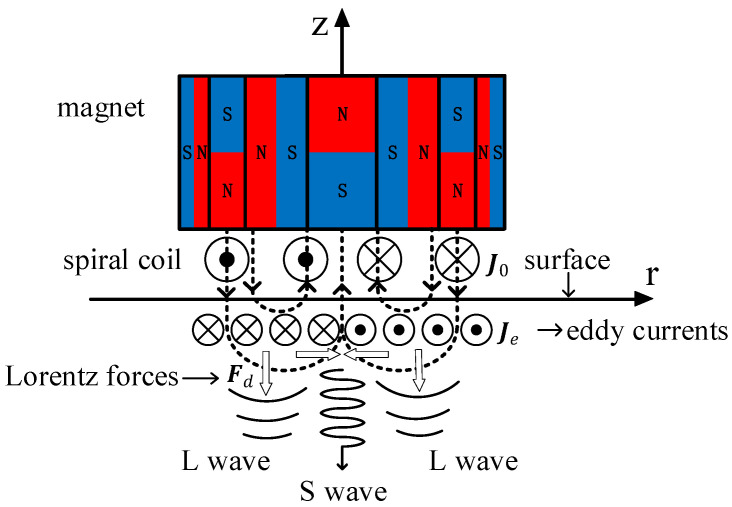
Schematic diagram of longitudinal wave and shear wave generation in EMAT.

The tested material used in this paper is aluminum, and in the non-ferromagnetic material, the EMAT excitation mechanism is the Lorentz force principle [26]. Figure 2 illustrates the generation of the longitudinal waves and shear wave in the non-ferromagnetic specimen. The white arrows in Figure 2 indicate the directions of Lorentz forces; the dotted lines indicate the direction of the magnetic field.

A spiral coil is placed on the surface of the test piece, and an excitation current $J_{0}$ is pulsed through the spiral coil. The high-frequency dynamic magnetic field $B_{d}$ will be induced in the test piece, and an eddy current $J_{e}$ with the same frequency as the current in the coil is also induced in the surface skin depth. Under the action of the static magnetic field $B_{s}$ and high-frequency dynamic magnetic field $B_{d}$, the particles on the specimen surface are subjected to the Lorentz forces $F_{s}$ and $F_{d}$. $F_{s}$ is the Lorentz force generated by the static magnetic field, and $F_{d}$ is the Lorentz force generated by the dynamic magnetic field. As a result, high-frequency periodic vibration occurs on the surface of the specimen, and elastic deformation is generated. Thus, an ultrasonic wave is produced, propagating in the specimen. The Lorentz force generated by the dynamic magnetic field $B_{d}$ and the static magnetic field $B_{s}$ on the specimen surface is [27]:

(1) $$F_{s}=J_{e}\times B_{s}$$

(2) $$F_{d}=J_{e}\times B_{d}$$

The total Lorentz force is:

(3) $$F_{L}=F_{s}+F_{d}$$

In the process of ultrasonic excitation, the in-plane dynamic magnetic field of EMAT is relatively small compared with the out-of-plane dynamic magnetic field [26]. Therefore, an approximate calculation of $F_{d}$ is made, and only the Lorentz force generated by the dynamic magnetic field in the *Z*-axis direction is considered. Considering that the static magnetic field from the magnet of the flux-concentrating EMAT has radial component $B_{sr}$ and axial component $B_{sz}$ into account, Equation (3) can be rewritten as:

(4) $$F_{s}=F_{r}^{\left(s\right)}+F_{z}^{\left(s\right)}$$

(5) $$F_{s}=J_{e}\times B_{sz}+J_{e}\times B_{sr}$$

where $F_{r}^{\left(s\right)}$ and $F_{z}^{\left(s\right)}$ are Lorentz forces in the radial direction and axial direction, respectively.

Under the action of the static radial magnetic field, the Lorentz force on the charged particles of the specimen surface is perpendicular to the specimen surface in the *Z*-axis direction and parallel to the propagation direction of the ultrasonic wave, thus generating a longitudinal wave. Under the action of the static axial magnetic field, the vibration direction of particles on the specimen surface is in the *R*-axis direction. In other words, the direction of the Lorentz force is perpendicular to the propagation direction of the ultrasonic wave, thus generating shear waves. Lorentz forces concentrate on the specimen surface and generate time-dependent elastic stress waves in the specimen. Consequently, the Lorentz forces $F_{r}^{\left(s\right)}$ and $F_{z}^{\left(s\right)}$ generate longitudinal waves and shear waves, respectively, both propagating in the thickness direction at the same time [19]. The process of the EMATs receiving the signal is the inverse process of their transmission. The reflected echo reaches the specimen surface, making the surface particles vibrate and changing the current in the spiral coil under the action of the static magnetic field.

## 3. Simulation Analysis

### 3.1. Dynamic Magnetic Field in Specimen

**Figure 3:**
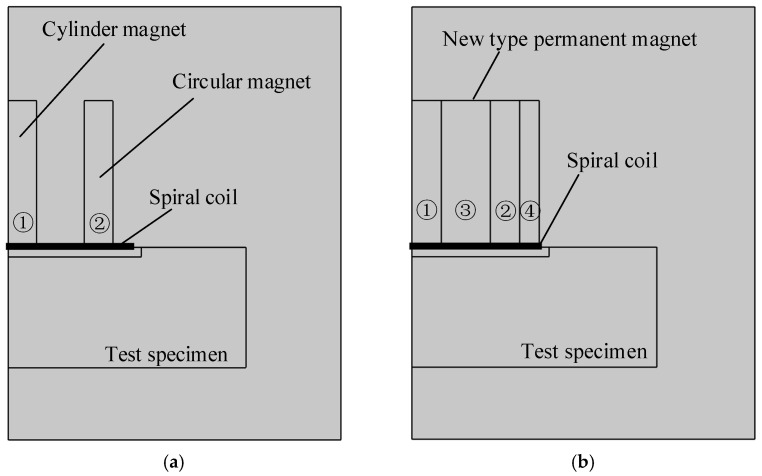
Model structure diagrams of (**a**) the ring-type longitudinal-wave EMAT and (**b**) the flux-concentrating longitudinal-wave EMAT. The No. 1 magnet is a cylinder magnet. The No. 2, No. 3 and No.4 magnets are circular magnets of different sizes.

**Figure 4:**
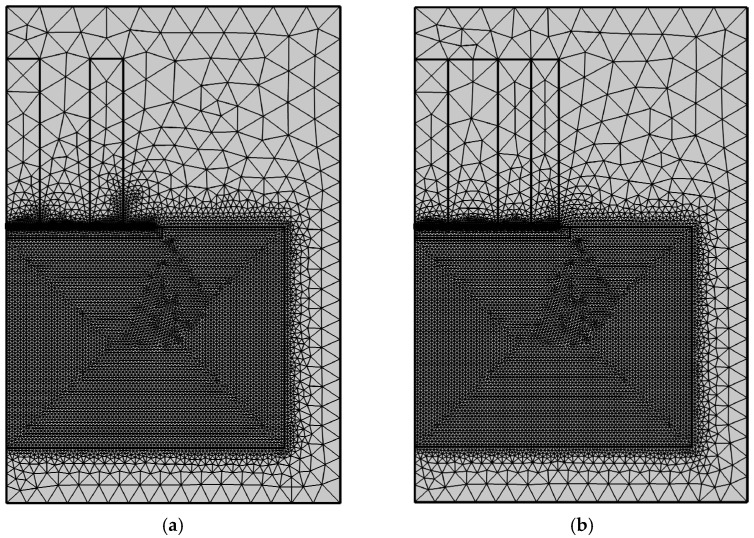
Meshes of (**a**) the ring-type longitudinal-wave EMAT model and (**b**) the flux-concentrating longitudinal-wave EMAT model.

Using the finite element software, COMSOL Multiphysics, two-dimensional axisymmetric solid simulation models are developed for the ring-type EMAT in Figure 1a and the flux-concentrating EMAT in Figure 1b, since they are all axisymmetric. Figure 3a,b shows the simulation model structures of the ring-type EMAT and the flux-concentrating EMAT with the left line as the symmetry axis, respectively. The parameters of magnets of the ring-type EMAT and flux-concentrating EMAT in finite element models are shown in Table 1. In models, the lift-off distance between the coil and the specimen is 0.2 mm, and the distance between the coil and the magnet is 0.3 mm. The right boundary of the 40 mm high aluminum specimen is set as a low reflection boundary to simulate the actual plate. The residual magnetic flux density of each permanent magnet is set as 1 T. By setting the frequency of the alternating current as 1 MHz, according to Equation (5), the Lorentz force is loaded into the Solid Mechanics Module to achieve the coupling of the electric, magnetic and elastic acoustic fields, so that the transmission of ultrasonic waves in the specimen can be observed in the time domain. Figure 4a,b shows the meshes of the two models. It should be noted that the maximum unit of the specimen mesh is set to 1/10 of the shortest wavelength to ensure the accuracy of the simulation.

**Figure 5:**
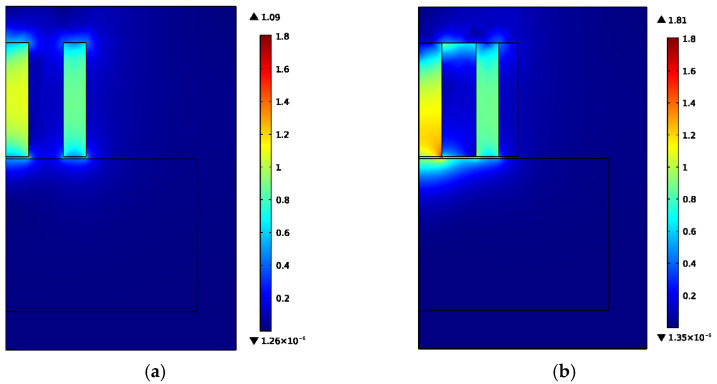
The magnetic flux density distribution in (**a**) the ring-type EMAT and (**b**) the flux-concentrating EMAT.

**Figure 6:**
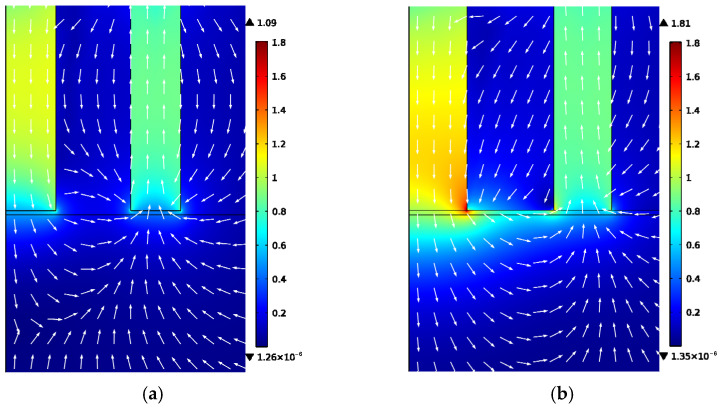
Local enlargement maps of (**a**) the ring-type EMAT and (**b**) the flux-concentrating EMAT.

The magnetic flux density distributions in the ring-type EMAT and the flux-concentrating EMAT are shown in Figure 5, and Figure 6 shows the partially enlarged views of the bottom of the magnets shown in Figure 5. The white arrow in Figure 6 shows the magnetic field direction of the permanent magnet. The magnetic field direction on the specimen surface under the No. 3 and No. 4 magnets is primarily horizontal, while the magnetic field direction under the No. 1 and No. 2 permanent magnets is primarily vertical.

**Figure 7:**
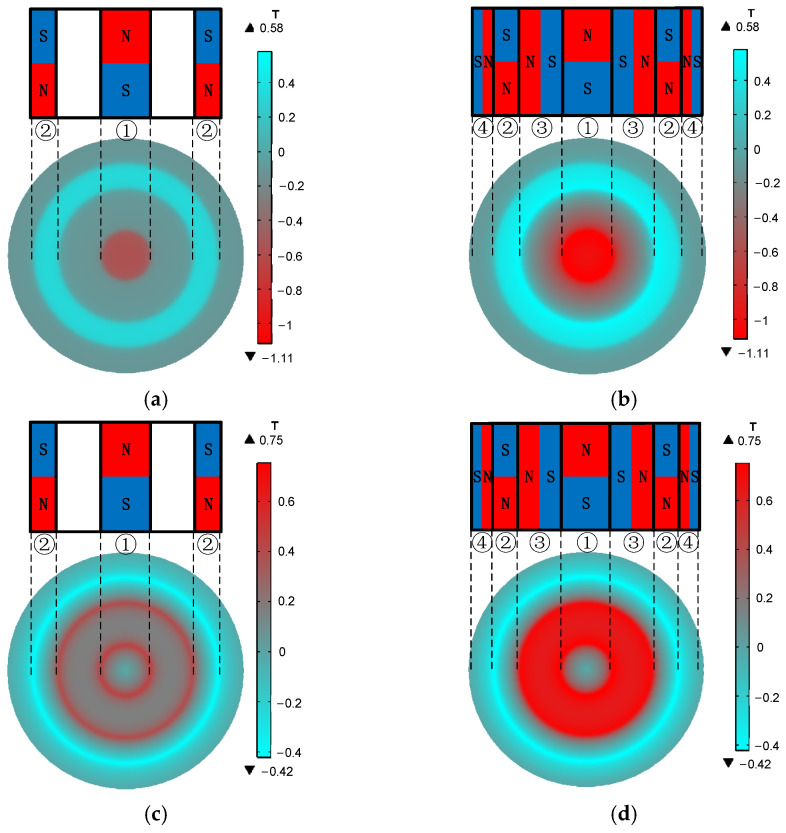
Magnet density distribution in a circular area with a radius of 30 mm from the center of the specimen surface. (**a**) Axial flux density component and (**c**) radial flux density component of the magnet in ring-type EMAT, (**b**) Axial flux density component and (**d**) radial flux density component of the magnet in flux-concentrating EMAT. The No. 1 magnet is a cylinder magnet. The No. 2, No. 3 and No.4 magnets are circular magnets of different sizes. The magnetizing directions of the magnets are shown.

**Figure 8:**
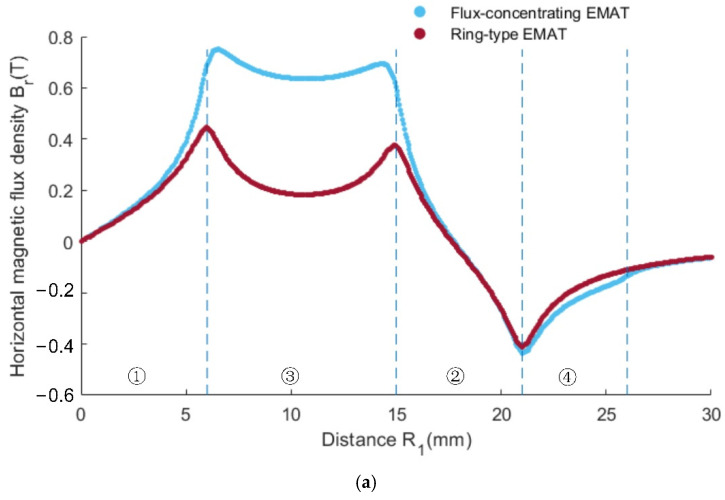

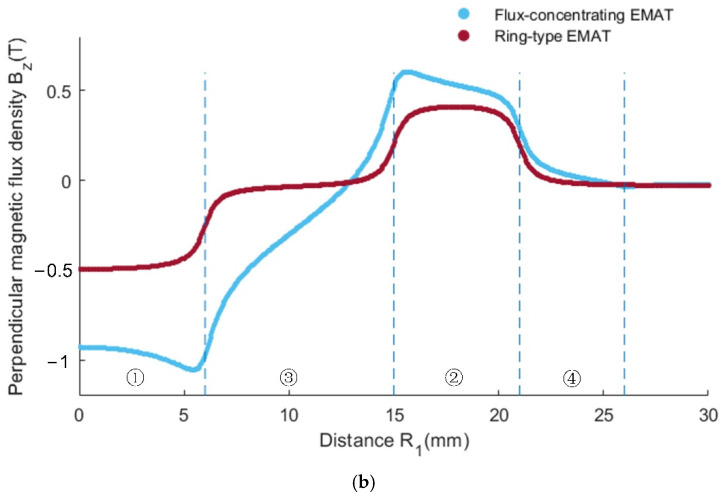
Magnetic flux profile of the ring-type EMAT and the flux-concentrating EMAT on specimen surface: (**a**) horizontal magnetic flux density, (**b**) perpendicular magnetic flux density.

It can be seen from Figure 7a,b that the axial component of the magnetic flux density of the ring-type EMAT and the flux-concentrating EMAT is mainly distributed in the area below the central cylinder magnet and No. 2 circular magnet. At these locations, the vertical flux density is greater than the horizontal flux density. The shear wave signals received by the coils in these locations have a higher amplitude. However, the vertical axial magnetic flux density of the flux-concentrating EMAT is higher than that of the ring-type EMAT so that the flux-concentrating EMAT can generate stronger shear waves. It can be seen from Figure 7c,d that the radial component of the magnetic flux density, which has a local maximum near the outer edge of the ring magnet, is mainly distributed in the area between the central cylinder magnet and No. 2 circular magnet. The horizontal flux density is greater than the vertical flux density in this area, where the longitudinal wave signals received by the coils have a higher amplitude. The horizontal radial magnetic flux density provided by the flux-concentrating EMAT occupies a larger part of the specimen surface, so that the flux-concentrating EMAT can generate stronger longitudinal waves. The comparison diagram of horizontal radial magnetic flux density $B_{sr}$ and perpendicular axial magnetic flux density $B_{sz}$ on the specimen surface is shown in Figure 8. As can be seen from the figure, the horizontal radial magnetic flux density of the flux-concentrating EMAT in the area from 6 mm to 16 mm from the center point on the specimen surface is significantly higher than that of the ring-type EMAT, which is about twice that of the ring-type EMAT. In addition, a strong vertical magnetic field is distributed in the circular region with a diameter of 12 mm in the center of the specimen surface to generate shear waves, which is about twice the vertical magnetic flux density of the ring-type EMAT. Therefore, the coils can be designed in different positions to generate shear waves or longitudinal waves.

### 3.2. Coil Design and Simulation of EMAT Signals

**Figure 9:**
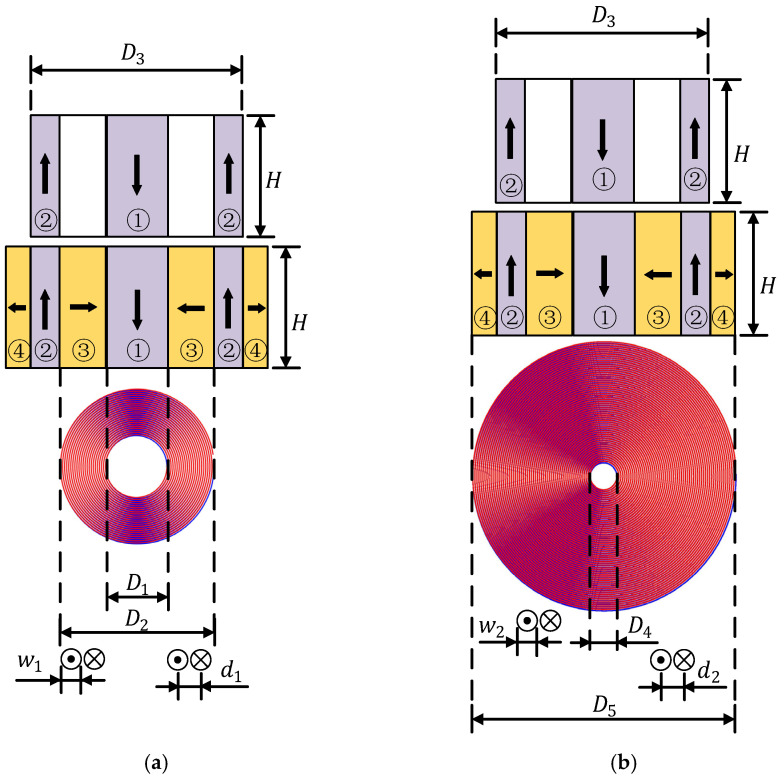
Top view of (**a**) L-mode coil and (**b**) dual-mode coil. The No. 1 magnet is a cylinder magnet. The No. 2, No. 3 and No.4 magnets are circular magnets of different sizes. The magnetizing directions of the magnets are shown.

**Figure 10:**
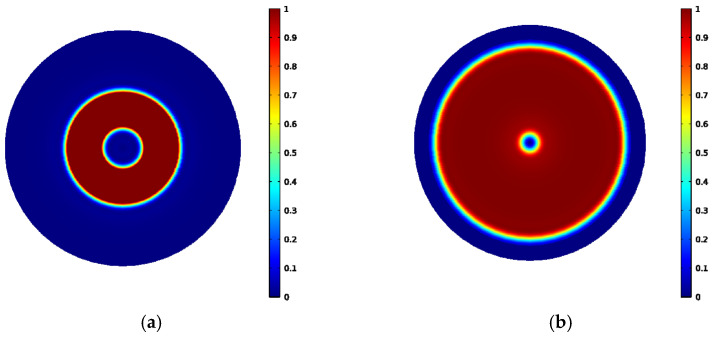
Eddy current distribution in a circular region with a radius of 30 mm from the center of the specimen surface: (**a**) L-mode coil, (**b**) dual-mode coil.

According to Figure 7 and Figure 8, we designed two kinds of coils with different sizes, shown in Figure 9. One is the L-mode coil that generates pure longitudinal waves, and the other is the dual-mode coil that can excite both longitudinal waves and shear waves. The coil size parameters are shown in Table 2. A high-frequency pulse current of 1 MHz is passed through the coil. The specimen below the coil induced eddy currents on the surface. Figure 10 shows the eddy current distribution on the specimen surface. The L-mode coil shown in Figure 9a, which can excite and receive relatively pure longitudinal waves, is in the radial magnetic flux density region. The diameter of the dual-mode coil, which is capable of exciting and receiving both longitudinal waves and shear waves, is equivalent to the overall diameter of the magnet.

**Figure 11:**
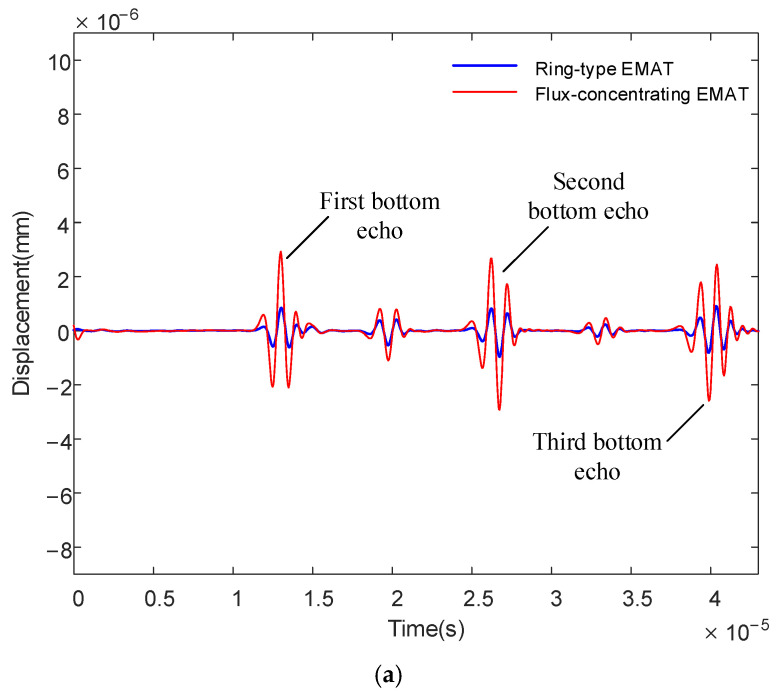

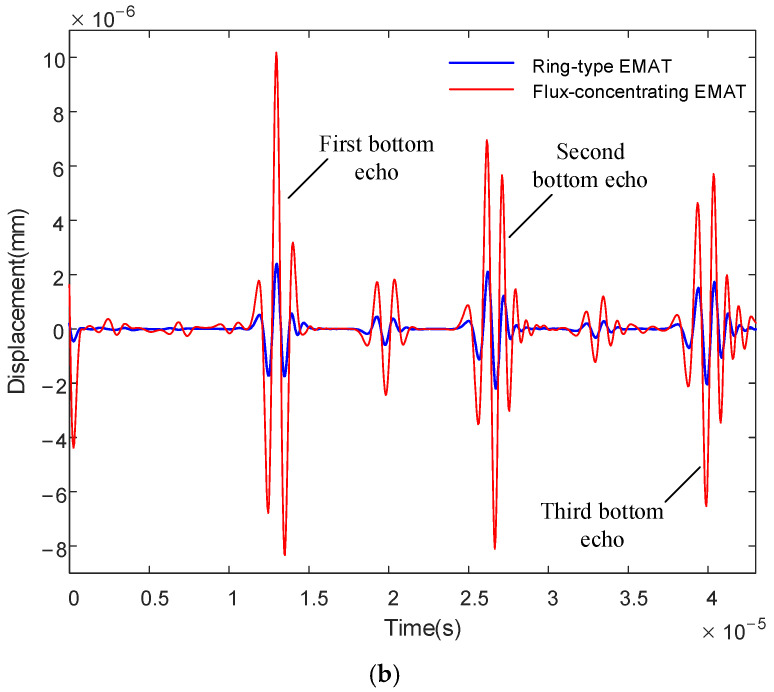
Average displacements of ring-type EMAT and flux-concentrating EMAT on the specimen surface (**a**) using dual-mode coil and (**b**) using L-mode coil.

Figure 11 shows the simulated average displacement of ring-type EMAT and flux-concentrating EMAT on the specimen surface as it changes over time. From the figure, we can see the simulated first bottom echo at 13.1 µs, the simulated second bottom echo at 26.2 µs and the simulated third bottom echo at 39.3 µs. The time interval between the two adjacent echoes is about 13 µs. The specimen in the model is aluminum with a thickness of 40 mm, in which the longitudinal wave propagation speed is 6100 m/s, and the shear wave propagation speed is 3050 m/s. Therefore, the theoretical peak time of the first longitudinal-wave echo is 13.1 µs. The theoretical peak time of the first shear wave echo is 26.2 µs and the theoretical peak time of the third bottom echo is 39.3 µs. The peak position of the simulated waveform is completely consistent with the theory. In Figure 11a,b, the displacement amplitudes of the flux-concentrating EMAT are all significantly higher than those of the ring-type EMAT no matter which coil is used. The first and third bottom echoes are mainly longitudinal waves, while the second bottom echo contains shear and longitudinal waves. Therefore, the flux-concentrating EMAT can generate stronger longitudinal waves than the ring-type EMAT. In addition, it is found from the simulation that there is an echo signal between the two adjacent echoes, which is generated by the mode conversion of longitudinal waves reflected on the surface or underside of the specimen.

**Figure 12:**
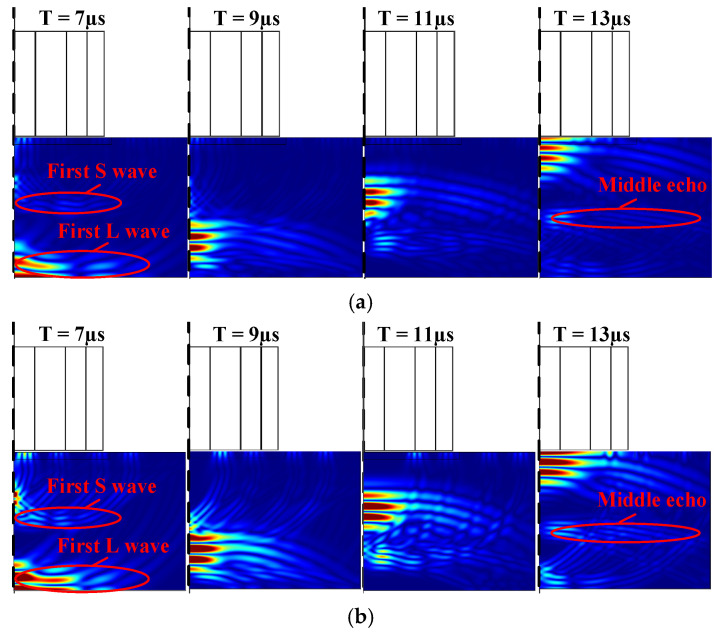

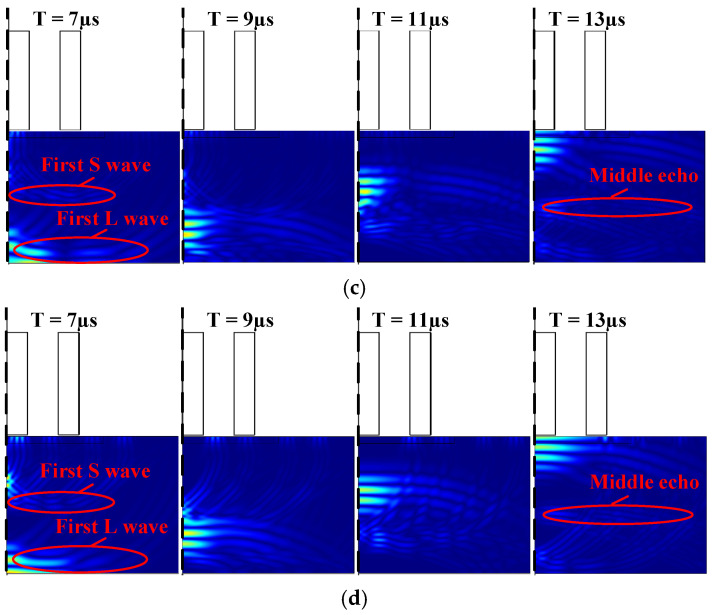
Total displacement propagation snapshots for (**a**) the flux-concentrating EMAT with dual-mode coil, (**b**) the flux-concentrating EMAT with L-mode coil, (**c**) the ring-type EMAT with dual-mode coil and (**d**) the ring-type EMAT with L-mode coil. The symmetry axis is the left dotted line.

To better show the mode conversion mechanism, the snapshots of the displacement with the same color scale are shown in Figure 12. Figure 12a–d refer to the flux-concentrating EMAT and ring-type EMAT working in dual and longitudinal mode, respectively. It is known that the velocity of a longitudinal wave is faster than that of a shear wave and the wavelength of the longitudinal wave is longer. Thus, according to the relative position and wavelength, all of the important waves related to Figure 11 in the snapshots are annotated. The middle echo, which appears after the first longitudinal wave reaches the bottom at 7 µs, is the shear wave according to the snapshots and propagating time.

## 4. Experiment

**Figure 13:**
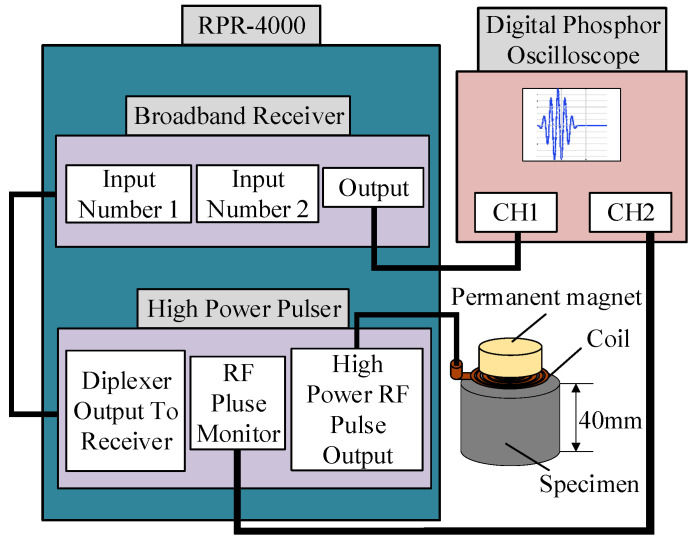
Experimental setup for measurement with EMATs.

**Figure 14:**
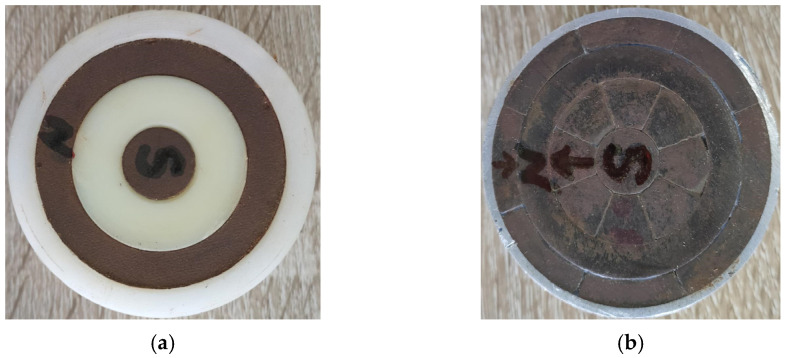
The fabrication of permanent magnets of (**a**) the ring-type EMAT and (**b**) the flux-concentrating EMAT.

**Figure 15:**
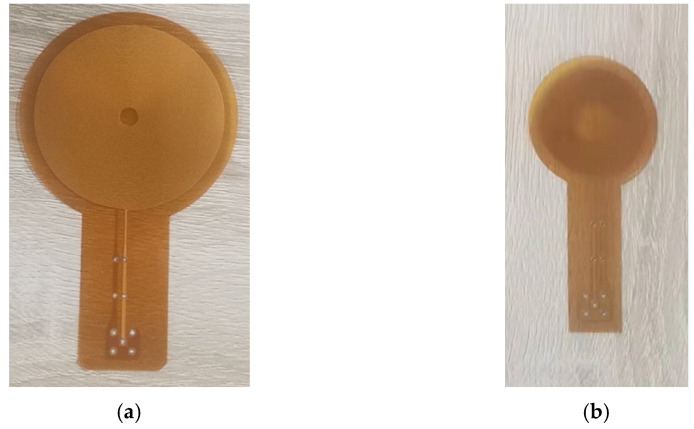
The fabrication of (**a**) dual-mode coil and (**b**) L-mode coil.

To verify the optimized performance of the flux-concentrating EMAT, a comparative experiment was conducted between the ring-type EMAT and the flux-concentrating EMAT. The schematic diagram of the experimental setup is shown in Figure 13. The magnets, combined with the spiral coil to simultaneously transmit and receive signals, were placed on top of the aluminum plate. The RPR-4000 pulse generator/receiver was chosen to excite a high-frequency pulse through the spiral coil in the experiment, whose duplex protected the preamplifier from the influence of the excitation pulse. The relevant parameters of the permanent magnet of the ring-type EMAT and the flux-concentrating EMAT are shown in Table 1, and the fabrication of the magnet and coil are shown in Figure 14 and Figure 15, respectively. What is worth mentioning is that the angular sector magnets of the flux-concentrating EMAT are hard to assemble because of the repulsive forces between adjacent magnets. The magnets need to be glued together, and the outside is secured with a metal ring. In addition, the center frequency of the excitation pulse was set as 1 MHz and the peak voltage of the excitation signal was up to 730 V. The test specimen was an aluminum plate with a thickness of 40 mm.

**Figure 16:**
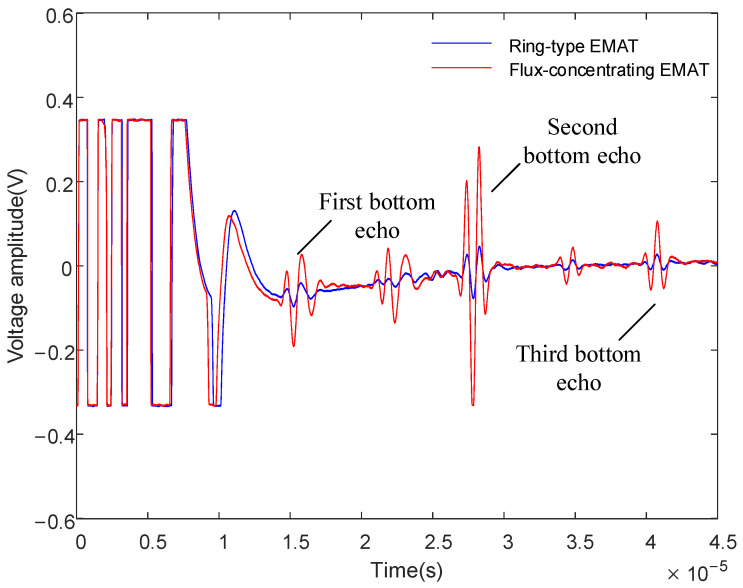
Signal diagram comparison of the ring-type EMAT and the flux-concentrating EMAT using the dual-mode coil.

The signal diagram, shown in Figure 16, of the dual-mode coil, which is shown in Figure 15a, demonstrates how a 40 mm thick aluminum plate was obtained. It can be seen that the time interval between the two bottom echoes measured by the ring-type EMAT and the flux-concentrating EMAT is about 13 μs, but the amplitude of the bottom echoes significantly increases. Compared with the amplitude of the signal received by the ring-type EMAT, the first bottom echo is increased by 276%, and the second bottom echo is increased by 391%.

The experimental results show that the flux-concentrating EMAT with the radial-flux-focusing magnet can simultaneously generate stronger longitudinal waves and shear waves. Figure 16 shows that the back echo signal of the flux-concentrating EMAT is easy to identify and has a high SNR. The first bottom echo is the longitudinal wave; the second bottom echo is a mixture of longitudinal waves and shear waves; and the third bottom echo is the longitudinal wave. In the signal diagram, we found a wave peak between two adjacent bottom echoes, which was concluded from the simulation, as the shear wave signal was generated by the wave mode transformation when the longitudinal wave reflected through the surface or bottom.

**Figure 17:**
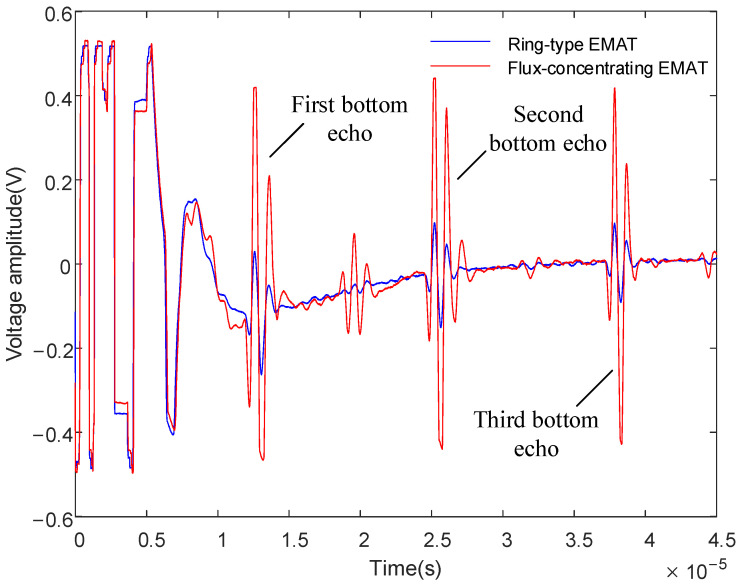
Signal diagram comparison of the ring-type EMAT and the flux-concentrating EMAT using the L-mode coil.

To suppress the generation of shear waves and obtain pure longitudinal waves, the L-mode coil shown in Figure 15b is used to measure the aluminum plate with a thickness of 40 mm. The comparison diagram of measurement signals of the ring-type EMAT and the flux-concentrating EMAT is shown in Figure 17.

From the longitudinal wave signal diagram shown in Figure 17, the L-mode coil can suppress the generation of shear waves and stimulate purer longitudinal waves with better signal recognition. Compared with the ring-type EMAT, the voltage amplitude of the first bottom echo of the received signal increases by 202%.

## 5. Conclusions

In this paper, a flux-concentrating EMAT composed of a radial-flux-focusing magnet is proposed. Based on the magnet of the ring-type EMAT, the radial-flux-focusing magnet is filled with a radial-magnetized annular magnet, which can provide a strong horizontal-radial magnetic field and a vertical axial magnetic field on the premise that the overall volume of the magnet does not change significantly. The ring-type EMAT with a similar structure and no magnetization is used for comparison.

Two-dimensional axisymmetric solid simulation models of the flux-concentrating EMAT and the ring-type EMAT operating on the non-ferromagnetic aluminum specimen were established to simulate the distribution of static magnetic flux. According to the simulation results, the flux-concentrating EMAT can provide a stronger horizontal radial magnetic field below the region of the No. 3 magnet and a vertical axial magnetic field in the central area, compared to the ring-type EMAT.

Based on theoretical and finite element analysis, two kinds of spiral coils with different sizes are designed: one is a longitudinal-wave EMAT, and the other is a dual-mode EMAT that can excite both longitudinal waves and shear waves. According to the simulation results, the flux-concentrating EMAT can improve the amplitude of the proposed wave mode significantly.

Finally, the ring-type EMAT and the flux-concentrating EMAT were used to measure the thickness of the 40 mm aluminum plate, and their received signals were compared. When the dual-mode coil is used, the voltage amplitude of the signal received by the flux-concentrating EMAT increases by 276% compared with the ring-type EMAT. When the L-mode coil is used, the voltage amplitude increases by 202%. It is verified that under the same pulse excitation, the received signal of the flux-concentrating EMAT is more accessible to identify than that of the ring-type EMAT. The voltage amplitude increases significantly, and the SNR is higher.

The flux-concentrating EMAT is not easy to assemble but has a higher energy conversion efficiency than the ring-type EMAT. Although the volume of the former is 63.7 cm^3^ and the volume of the latter is only 41.6 cm^3^, provided that the same static magnetic field strength is needed, the flux-concentrating structure could be combined with more economical magnets of lower energy products, and different coils according to the distribution of magnet direction could be used to achieve dual-mode excitation or pure longitudinal wave excitation according to specific needs. For pure longitudinal wave excitation, the shear wave signal is suppressed while the longitudinal wave signal is strengthened in resonant ultrasound spectroscopy, thus the interference of shear waves to longitudinal waves is greatly reduced. The designed EMAT could be applied to increase the detection speed in the field of thickness measurement. For dual-mode excitation, the flux-concentrating EMAT could have similar applications in elastic-constant extraction, which is consistent with other similar studies [20,24]. Moreover, the cylindrical-design EMAT are fit for measuring the axial stress of non-ferromagnetic cylinder-like aluminum bolts, and we hope to conduct further research on this topic in the future.

## Figures and Tables

**Table 1:** Parameters of EMAT magnet used in this paper.

Magnet Number	Magnet Parameters	Symbol	Value (mm)
1	Diameter	$D_{1}$	12
	Height	H	30
2	Inner diameter	$D_{2}$	32
	Outer diameter	$D_{3}$	44
	Height	H	30
3	Inner diameter	$D_{1}$	12
	Outer diameter	$D_{2}$	32
	Height	H	30
4	Inner diameter	$D_{1}$	44
	Outer diameter	$D_{2}$	52
	Height	H	30

**Table 2:** Size parameters of L-mode coil and dual-mode coil.

Coil Type	Size Parameters	Symbol	Value (mm)
L-mode coil	Inner diameter	$D_{1}$	12
	Outer diameter	$D_{2}$	32
	Wire width	$w_{1}$	0.2
	Wire spacing	$d_{1}$	0.2
Dual-mode coil	Inner diameter	$D_{4}$	5
	Outer diameter	$D_{5}$	52
	Wire width	$w_{2}$	0.2
	Wire spacing	$d_{2}$	0.4
